# A SEER database retrospective cohort of 547 patients with penile non-squamous cell carcinoma: demographics, clinical characteristics, and outcomes

**DOI:** 10.3389/fonc.2023.1271913

**Published:** 2023-10-31

**Authors:** Lucas W. Ashley, Kent F. Sutton, Andrew Ju, George Edwards, Melisa Pasli, Arjun Bhatt

**Affiliations:** ^1^ Brody School of Medicine, Greenville, NC, United States; ^2^ Duke University, Durham, NC, United States; ^3^ Department of Radiation Oncology, ECU Health, Greenville, NC, United States

**Keywords:** penile cancer, histology, surgery, treatment trends, SEER

## Abstract

**Introduction:**

Little research has investigated the prevalence and distribution of the diverse pathologies of non-squamous cell carcinoma (non-SCC) of the penis. Although rare in clinical practice, these cancers have become a focus of greater importance among patients, clinicians, and researchers, particularly in developing countries. The principal objective of this study was to analyze the major types of penile non-SCC, elucidate common treatment pathways, and highlight outcomes including 5-year survival.

**Materials/methods:**

The Surveillance, Epidemiology, and End Results (SEER) database was queried between 2000 and 2018 to identify a retrospective cohort of patients with penile non-SCC. Demographic information, cancer characteristics, diagnostic methods, treatments administered, and survival were investigated.

**Results:**

A total of 547 cases of penile non-SCC were included in the analysis. The most prevalent non-SCC cancers included epithelial neoplasms, not otherwise specified (NOS) (15.4%), unspecified neoplasms (15.2%), basal cell neoplasms (13.9%), blood vessel tumors (13.0%), nevi and melanomas (11.7%), and ductal and lobular neoplasms (9.9%). Over half (56.7%) of patients elected to undergo surgical intervention. Patients rarely received systemic therapy (3.8%) or radiation (4.0%). Five-year survival was 35.5%. Patients who underwent surgery had greater annual survival for 0–10 years compared to those who did not have surgery. Significant differences in survival were found between patients who had regional, localized, and distant metastases (*p* < 0.05). A significant difference in survival was found for patients married at diagnosis versus those who were unmarried at diagnosis (*p* < 0.05). Lower survival rates were observed for patients older than 70 years.

**Discussion:**

Although less prevalent than SCC, penile non-SCC encompasses a diverse set of neoplasms. Patients in this cohort had a high utilization of surgical management leading to superior outcomes compared to those not receiving surgery. Radiation is an uncommonly pursued treatment pathway. Patient demographics and socioeconomic variables such as marital status may be valuable when investigating cancer outcomes. This updated database analysis can help inform diagnosis, management, and clinical outcomes for this rare group of malignancies.

## 1 Introduction

Penile cancer is a rare malignancy that is estimated to affect 2,050 males and cause 470 deaths in the United States (U.S.) in 2023 alone (1). While these cancers are uncommon in the U.S., patients living in the continents of South America, Africa, and Asia are at higher risk. In these locations, penile cancer can account for up to 20% of all malignancies in males (2). As these populations are rarely included in research studies, any potentially clinically relevant data from American populations could be valuable in the appropriate context, thus, such research may be of understated importance (3). Much of the current understanding of penile cancer comes from global databases, and this work suggests that penile cancer is staying constant, as in Germany, France, Denmark, and Norway, if not increasing in prevalence in faster growing nations such as Thailand, Nigeria, and India (4–6). Country-specific efforts to understand the epidemiology of penile cancer tend to emphasize psychosocial and cultural factors related to disease, including practices related to circumcision, genital hygiene (e.g., smegma clearance), the impacts on patient identities following diagnosis, and the tracking of human papillomavirus (HPV); while prognostic and therapeutic-oriented research continues to make steady but slow progress toward the development of treatment guidelines, significant controversy over best practices still remain (7–10).

Over 95% of penile cancers have squamous cell carcinoma (SCC) histology, including virtually all penile cancers associated with HPV and, thus, these cancers have been the main focus of research on penile cancer thus far (11). The remaining cases are classified as non-SCC, consisting of a wide range of histologies, including adenocarcinoma, melanoma, basal cell carcinoma, lymphomas, and soft tissue sarcomas (12, 13). While penile non-SCC accounts for a small proportion of all penile cancers, little research has investigated the prevalence, distribution, and characteristics of its diverse etiologies.

Previous analyses of patients with non-SCC of the penis examined cohorts with characteristics that may limit the generalizability of their results. Of the 666 patients identified in a previous study, all patients diagnosed with penile non-SCC between 1975 and 2016 were included in their analyses, but a substantial portion (42.2%) was diagnosed prior to 2000 (14). As a result, the authors’ results may not represent the current histological trends nor the influences of current therapy with regard to prognosis and eventual outcome for penile non-SCC malignancies. For example, the incidence of penile Kaposi’s sarcoma (KS) has decreased dramatically since that time with the advent of antiretroviral medication. While this work is the first large-scale study of the topic, it did not exclude patients with precursor lesions, which are now better appreciated to be pathologically distinct from the invasive cancers of the present examination (15). Another analysis, in contrast, used the SEER database to consider a more recent cohort; however, this study does not include patients diagnosed in the years since 2016 and, as a consequence of the way their study design queried histology with defined subtypes, did not include some of the rarer subtypes of non-squamous penile cancer. Consequently, this study consists of a relatively small sample size of 123 patients with penile non-SCC (15). International literature on non-SCC of the penis is even more limited, consisting mainly of case reports (16–19). These considerations highlight the need for additional studies investigating the changing landscape of these understudied malignancies.

The rarity of penile non-SCC creates obstacles to conducting large-scale cohort studies; one downstream effect of this is that there is a deficit of guidelines for treatment and providing patient prognoses. Our study aims to provide recent histological trends of penile non-SCC as well as elucidate common treatment pathways and highlight significant survival outcomes. Here, we provide an update that more than triples the sample size of the most recent SEER study while including subgroup analyses of histological types as they pertain to lymph node biopsy and trends in their clinical Stage at presentation.

## 2 Methods

### 2.1 Data source and cohort selection

The Surveillance, Epidemiology, and End Results (SEER) database of the National Cancer Institute, which encompasses approximately 48% of the U.S. population, was queried to identify a retrospective cohort (20). A total of 547 patients diagnosed with penile non-SCC between the years 2000 and 2018 were identified using the International Classification of Disease for Oncology Version 3. Patients were identified using the site code C60, which corresponds to primary malignancy of the penis. All patients with SCC histology codes (8050–8089) were excluded, as were all patients with benign lesions on or metastases to the penis. All included patients were at least 15 years of age at the time of diagnosis and had no other diagnoses of malignancy.

### 2.2 Data processing

For survival analyses, patients without numeric values (e.g., “Unknown”) encoded for their survival in months were omitted. For Stage analyses, SEER Summary Staging definitions were cross-referenced with National Comprehensive Cancer Network (NCCN) staging guidelines. SEER Summary Staging of “Local” corresponded to Stage T1a or T1b, “Regional” corresponded to T2 or T3 or N1 to N3, and “Distant” corresponded to T4 or M1 (21) (22). Precise translation between SEER and NCCN staging was not strictly possible and does represent a limitation of the present study; however, the SEER dataset was found to be internally consistent with respect to Stage variables and variables such as “Lymph Node Examined,” “Lymph Node Positive,” and variables related to metastatic disease. Lymphomatous disease staging was encoded separately in SEER and corresponded with the Ann Arbor staging schema.

### 2.3 Statistical analysis

Data obtained from the SEER database were analyzed utilizing IBM SPSS Statistics for Windows, Version 27.0 (Armonk, NY: IBM Corp). The sample was characterized using descriptive statistics, namely, means, medians, percentages, and frequencies. Survival of the cohort over a 10-year period was assessed using the Kaplan–Meier estimator, and statistical significance was assessed using the log-rank test. For comparisons of prevalence, a one-sided t-test was used. A *p*-value < 0.05 was deemed statistically significant for all analyses. Confidence intervals (CI) were set at 95%.

Covariates selected for the analysis included multicategory variables such as race, tumor histology, and SEER Summary Stage, for which each value of the variable was compared to every other value as well as the cohort at large, and variables parsed as binary variables such as age at diagnosis (younger or older than the median of 70 years), marital status (married vs. not), surgery (received vs. not), any regional lymph nodes examined (Yes/No), any regional lymph nodes positive (Yes/No), radiation received (Yes/No), and systemic therapy received (Yes/No). We attempted to include covariates regarding the extent of disease and lymph node surgical removal; however, 80% or more of the data was missing for each variable, thus limiting this mode of analysis. Multivariate analysis was conducted using a Cox Proportional Hazards model, as implemented by Python package lifelines.

### 2.4 Ethical considerations

This study is exempt from ethical review because the SEER database does not include identifying information of patients in the cohort.

## 3 Results

### 3.1 Patient and tumor characteristics

Between the years 2000 and 2018, the SEER database yielded 547 cases of non-SCC of the penis in accordance with our inclusion criteria.

The patients in this cohort had an average age of 65.6 (SD = 15.1) years and an age range of 15–90 years. 100% of the patients were male. A majority of the patients were White (79.0%), followed by Asian or Pacific Islander (9.3%), Black (8.2%), and American Indian/Alaska Native (1.5%).

Nearly half (48.1%) of the cohort was married (including common law marriage). The remaining patients (36.1%) whose status was known were categorized as unmarried. These patients, however, fall into various relationship status categories such as divorced, separated, single (never married), unmarried or in a domestic partnership, or widowed. Complete patient demographics are shown in **Table 1**.

**Table 1:** Patient demographics, median OS, and 5-year survival.

Patient demographics	*n* (%)	Median OS (in months)	5-year survival (%)
Age, mean (SD)	65.6 (15.1)	38.5 (33.4.6–43.6)	37.7
< 70	266 (48.6)	65.0 (62.8–72.7)	57.3
≥ 70	281 (51.4)	27.0 (21.0–33.0)	23.8
Sex			
Male	547 (100)	38.5 (33.4–43.6)	37.7
Female	0 (0)	–	–
Race			
White	432 (79.0)	39.0 (33.2–44.8)	37.5
Asian or Pacific Islander	51 (9.3)	60.0 (45.4–74.6)	50.0
Black	45 (8.2)	19.0 (2.83–35.2)	23.1
American Indian/ Alaska Native	8 (1.5)	4.0 (0–8.7)	0
Marital status			
Married	263 (48.1)	47.0 (39.0–55.0)	43.3
Unmarried	203 (37.1)	16.0 (8.0–24.0)	28.1
Surgery received?			
No	179 (32.7)	13.0 (5.27–20.7)	25.1
Yes	321 (58.6)	50.0 (43.5–56.5)	44.9

The most prevalent histologies among penile non-SCC included epithelial neoplasms, NOS (15.4%), unspecified non-epithelial neoplasms (15.2%), basal cell neoplasms (13.9%), blood vessel tumors (13.0%), melanomas (11.7%), and ductal and lobular neoplasms (9.9%). Blood vessel tumors included KS (11.7%). 49.7% of neoplasms were classified as carcinomas of the penis. Less prevalent cancers include transitional cell carcinoma, adenocarcinoma, and myomatous neoplasms. Median overall survival (OS) and 5-year survival for the tumor histological groupings are shown in **Table 2**.

**Table 2:** Tumor characteristics.

Tumor characteristics	*n* (%)	Median OS in months (95% CI)	5 year survival (%)
Tumor histology			
Epithelial neoplasms, NOS Unspecified neoplasms Basal cell neoplasms Blood vessel tumors Melanomas Ductal and lobular neoplasms Transitional cell papillomas and carcinomas Myomatous neoplasms Adenocarcinomas NHL - mature b-cell lymphomas Adnexal and skin appendage neoplasms Soft tissue tumors and sarcomas, NOS NHL - mature T- and NK-cell lymphomas Fibromatous neoplasms Complex epithelial neoplasms Complex mixed and stromal neoplasms Cystic, mucinous and serous neoplasms Mesothelial neoplasms Malignant lymphomas, NOS or diffuse Plasma cell tumors	84 (15.4) 83 (15.2) 76 (13.9) 71 (13.0) 64 (11.7) 54 (9.9) 27 (4.9) 17 (3.1) 16 (2.9) 12 (2.2) 9 (1.6) 9 (1.6) 7 (1.3) 6 (1.1) 4 (0.7) 3 (0.5) 2 (0.4) 1 (0.2) 1 (0.2) 1 (0.2)	8.0 (0–19.0) 6.0 (0–15.9) 53.0 (40.2–65.8) 76.0 (59.4–92.6) 52 (37.3–66.7) 67.5 (52.9–82.1) 26.0 (12.2–39.8) 56.0 (37.9–74.1) 16.0 (0–43.2) 26.0 (0–62.1) 94.0 (40.3–147.7) 19.0 (2.3–35.7) 104.0 (42.2–165.8) 4.5 (0–33.6) 8.0 (4.4–11.6) 19.0 (9–66.9) 28.0 (0–59.4) 110.0 143.0 21.0	18.75 13.0 43.4 57.7 42.2 53.7 18.5 47.1 37.5 33.3 66.7 11.1 71.4 16.7 0 33.3 0 100 100 0
Tumor stage			
Localized disease Regional disease Distant disease Unknown/Unstaged	225 (41.1) 61 (11.2) 30 (5.5) 91 (16.6)	51.0 (44.7–57.3) 19.0 (9.84–28.2) 4.0 (0.45–7.5) 14.0 (6.81–21.2)	45.8 14.8 0 19.8
Tumor grade			
B cell; pre-B cell; B-cell precursor T cell Well differentiated; grade I Moderately differentiated; grade II Poorly differentiated; grade III Undifferentiated; anaplastic; grade IV Unknown	9 (1.6) 9 (1.6) 9 (1.6) 20 (3.7) 55 (10.1) 17 (3.1) 372 (68.0)	29.0 (0–72.3) 104.0 (70.9–137.1) 82.0 (44.2–119.8) 56.0 (34.3–77.7) 17.0 (6.23–27.8) 19.0 (0–45.5) 45.0 (38.9–51.1)	44.4 66.7 77.8 45.0 21.8 23.5 40.1

### 3.2 Treatment

#### 3.2.1 Surgery

Three hundred twenty-one (58.7%) patients underwent surgery, while 179 (32.7%) did not have surgery. For 57 (8.6%) participants, it is unknown whether surgery was performed. There was a wide variety of surgical techniques used for treatment. Most patients underwent excisional biopsy (24.5% of all patients), followed by simple or partial removal of the primary site tumor (15.0%), wide local tumor excision, NOS (8.6%), and total surgical removal of the tumor primary site including penectomy (5.3%). Approximately 92 patients received surgery that included lymph node dissection (29% of all patients who received surgery); positive lymph nodes were found in 20 of those cases. The histologies most often associated with positive lymph nodes were melanomas, transitional cell papillomas and carcinomas, and unspecified epithelial tumors; the three most overrepresented histologies regarding positive lymph nodes as a proportion of frequency were transitional cell papillomas and carcinomas (18.5%), nevi and melanomas (14.1%), and soft tissue tumors and sarcomas (11.1%). Full results for histologies and rates at which cancer was found in lymph nodes for each histology are presented in **Table 3**.

**Table 3:** Histologies, stage at diagnosis, and comparison of LN surveyed and LN+.

Histology		Stage			Lymph nodes		
	*N* (%)	Local	Regional	Distant	LN examination	LN+	LN hit rate
Epithelial neoplasms, NOS	84 (15.4)	23.5	20.6	11.8	6	5	0.8333333333
Unspecified neoplasms	83 (15.2)	8.8	8.8	8.8	3	2	0.6666666667
Basal cell neoplasms	76 (13.9)	78.9	1.8	3.5	1	0	0
Blood vessel tumors	71 (13.0)	75	9.6	0	3	2	0.6666666667
Melanomas	64 (11.7)	58.3	27.1	4.2	35	9	0.2571428571
Ductal and lobular neoplasms	54 (9.9)	80.4	6.5	2.2	2	1	0.5
Transitional cell papillomas and carcinomas	27 (4.9)	37.5	29.2	16.7	6	5	0.8333333333
Myomatous neoplasms	17 (3.1)	78.6	0	7.1	1	0	0
Adenocarcinomas	16 (2.9)	72.7	9.1	9.1	2	1	0.5
NHL - mature b-cell lymphomas	12 (2.2)	57.1	28.6	14.3	0	0	
Adnexal and skin appendage neoplasms	9 (1.6)	87.5	0	0	0	0	
Soft tissue tumors and sarcomas, NOS	9 (1.6)	55.6	33.3	11.1	2	1	0.5
NHL - mature T- and NK-cell lymphomas	7 (1.3)	85.7	14.3	0	0	0	
Fibromatous neoplasms	6 (1.1)	0	0	3	1	0	0
Complex epithelial neoplasms	4 (0.7)	0	66.7	0	0	0	
Complex mixed and stromal neoplasms	3 (0.5)	66.7	33.3	0	1	0	0
Cystic, mucinous and serous neoplasms	2 (0.4)	50	50	0	0	0	
Mesothelial neoplasms	1 (0.2)	0	0	0	0	0	
Malignant lymphomas, NOS or diffuse	1 (0.2)	100	0	0	0	0	
Plasma cell tumors	1 (0.2)	0	100	0	0	0	

#### 3.2.2 Adjuvant therapy

Patients rarely received adjuvant therapies. Forty-three (7.9%) patients received systemic therapy and 43 received radiation. Thirteen (2.4%) underwent both systemic therapy and radiation. Of the 21 patients (3.8%) that received systemic therapy in addition to surgery, 18 (3.3%) did so after surgery, two (0.37%) did so before surgery, and one (0.18%) did so both before and after surgery. Of the 22 patients (4.0%) that received radiation in addition to surgery, 19 (3.5%) did so after surgery, one (0.18%) did so prior to surgery, and two (0.37%) received both radiation and surgery, but the sequence is unknown. Multivariate analysis revealed a higher concordance between disease Stage and the receipt of adjuvant chemotherapy than disease grade and adjuvant chemotherapy (**Supplementary Table S1**).

#### 3.2.3 Definitive radiation

**Figure 1:**
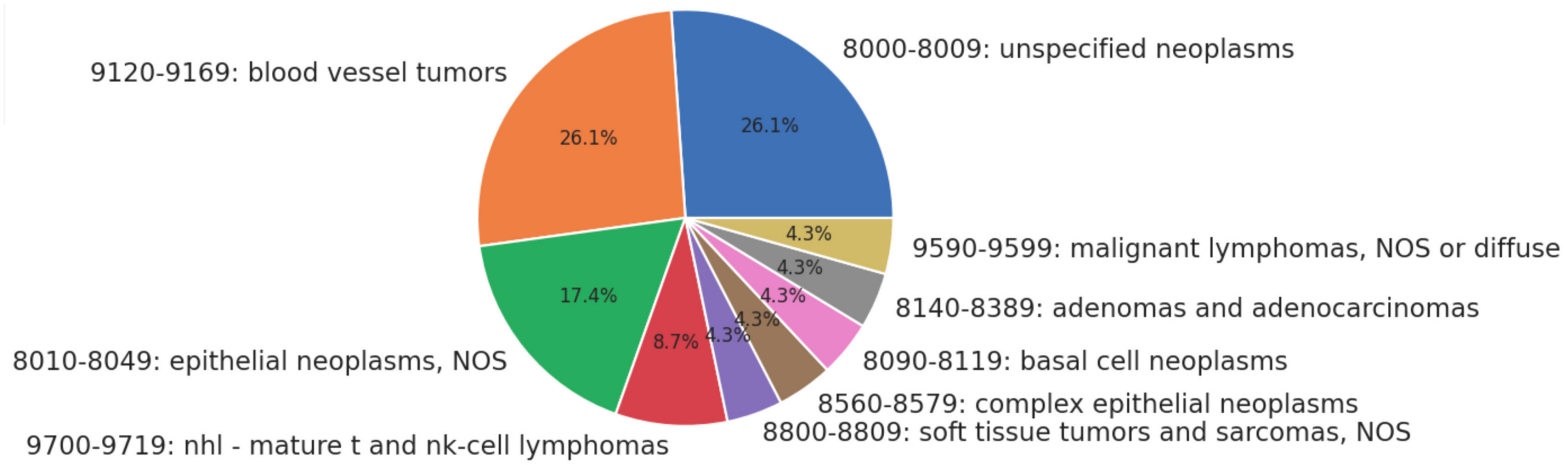
Histologies treated with definitive radiotherapy.

**Figure 2:**
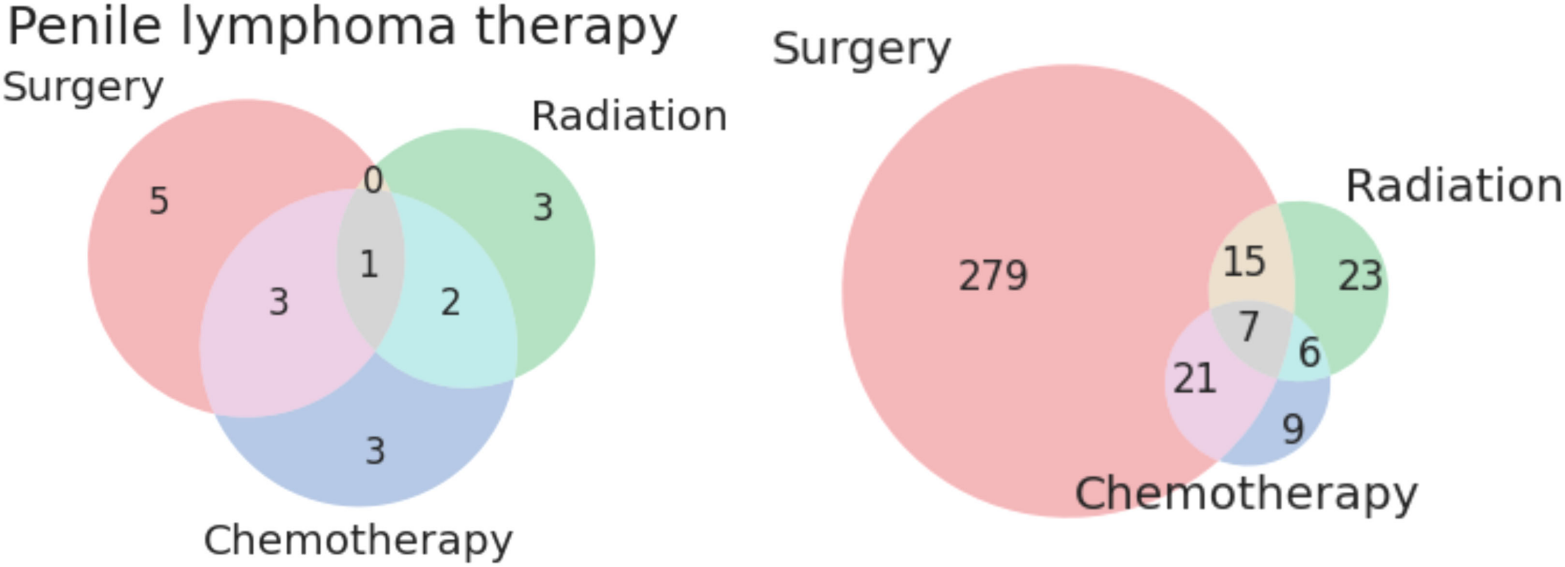
Venn diagrams of treatment modalities for lymphomatous (left) and non-lymphomatous non-squamous tumors (right) of the penis.

Twenty-three patients received only radiation as treatment, receiving no surgery or chemotherapy. None of the patients in this category had metastases or advanced disease Stage. The distribution of histologies for patients treated solely with radiation is presented as **Figure 1** below. The most common treatment modalities and combinations thereof are presented in **Figure 2**; lymphomas were evaluated separately.

### 3.3 Survival

**Figure 3:**
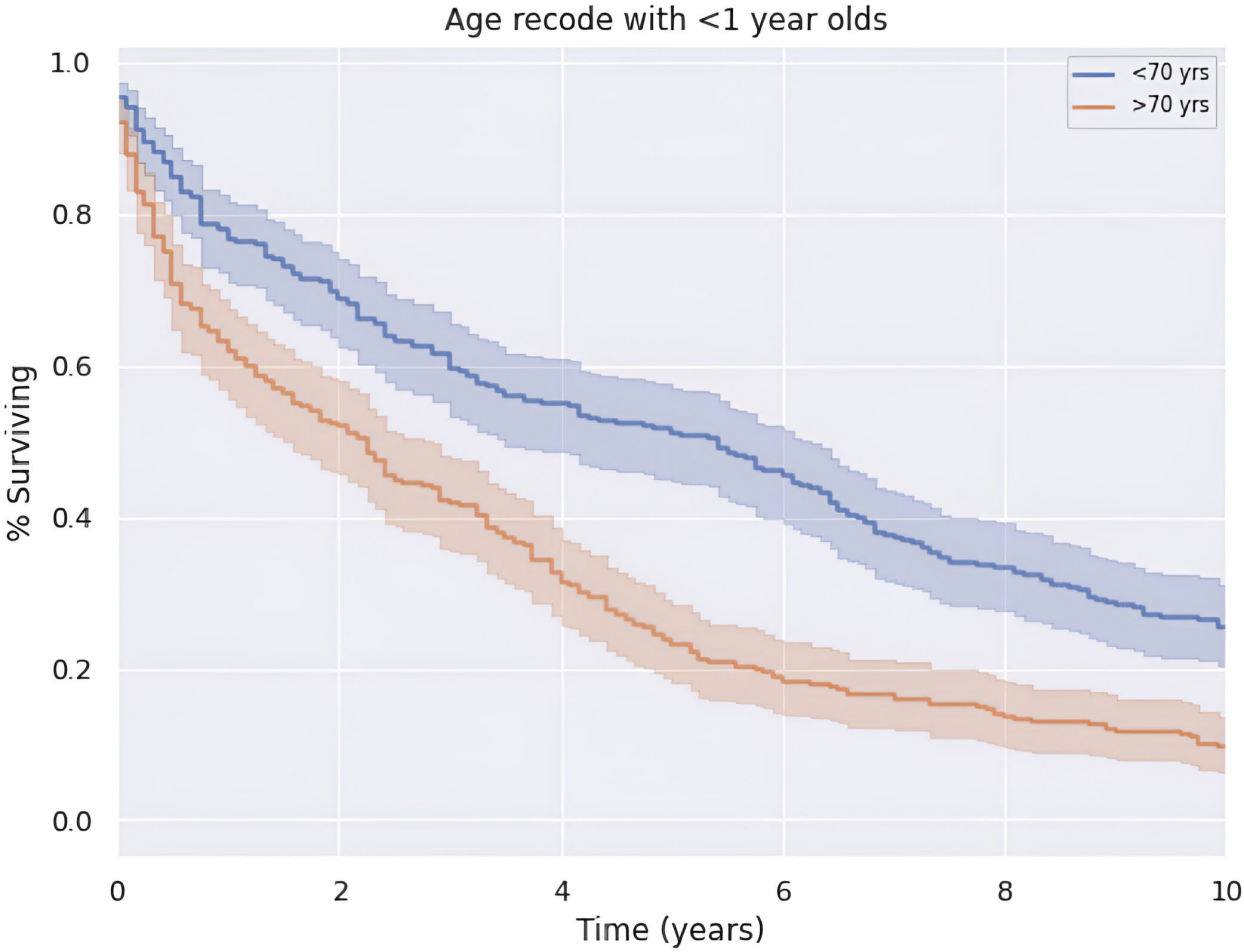
Survival versus age at diagnosis.

Median OS of the entire cohort was 3.2 years or 38.5 months (CI: 33.4–43.6). Five-year survival was 37.7%. There is a greater 5-year OS in patients less than the median age of 70 years old compared to those older than 70 years old (*p* < 0.05) as shown in **Figure 3**. Patients younger than 70 years old had a median OS and 5-year survival rate of 65.0 months (CI: 62.8–72.7) and 57.3%, respectively. Alternatively, patients at 70 years of age or older had a median OS and 5-year survival rate of 27.0 months (CI: 21.0–33.0) and 23.8%, respectively.

**Figure 4:**
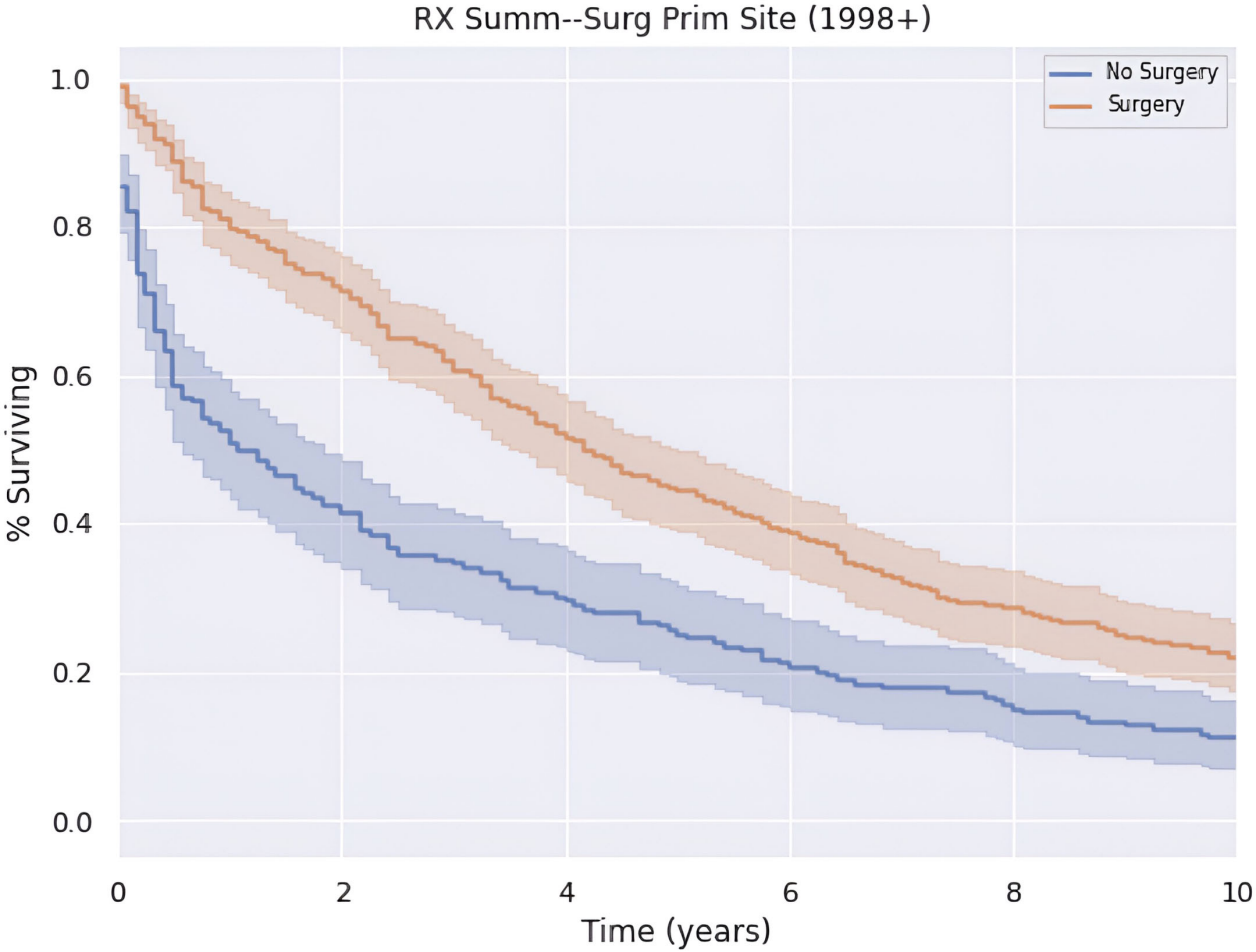
Impact of primary tumor site surgery on survival.

Patients who underwent surgery had greater annual survival compared to those who did not undergo surgery (*p* < 0.05), as shown in **Figure 4**. Those who received surgery had a median OS and 5-year survival of 50.0 (CI: 43.5–56.5) months and 44.9%. In contrast, patients in this cohort who did not undergo surgery had a median OS and 5-year survival of 13.0 (CI: 5.27–20.7) months and 25.1%.

**Figure 5:**
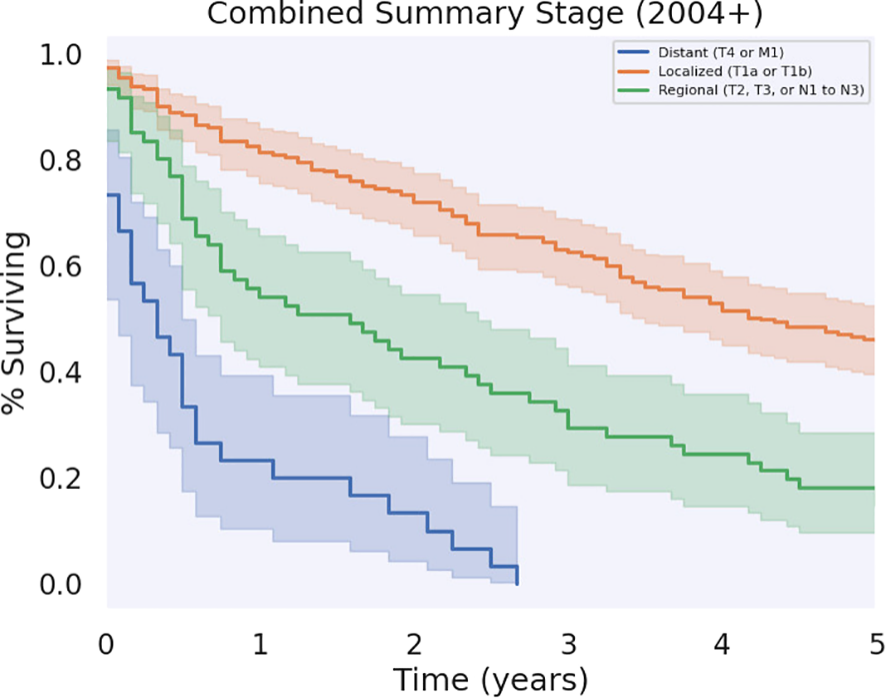
Survival by disease stage.

Significant differences in survival were found between patients who had regional, localized, and distant metastases (*p* < 0.05), as shown in **Figure 5**. Patients who had localized disease had the greatest annual survival, with a median OS of 51 months (CI: 44.7–57.3). Those who had regional disease had decreased annual survival compared to localized disease, greater annual survival with respect to distant disease, and a median OS of 19.0 (CI: 9.84–28.2). Patients with distant disease had the poorest survival rates across the cohort, with a median OS of 4.0 (CI: 0.45–7.5). The 5-year survival for localized, regional, and distant metastases were 45.8%, 14.8%, and 0%, respectively. Unknown and unstaged disease, referring to cases without a Stage recorded by SEER, had a median OS of 14.0 (CI: 6.81–21.2) and 5-year survival of 19.9%.

A large majority (78.2%) of patients have tumors of unknown grade. For these patients, the information was missing in the database or classified as unknown. Fifty-five (10.1%) patients had tumors that are poorly differentiated with the classification of grade III. Twenty (3.7%) patients had moderately differentiated or grade II tumors, and nine (1.6%) patients had well differentiated or grade I tumors. Additionally, nine (1.6%) patients had B-cell grade tumors, and nine patients had T-cell grade tumors. Median OS and 5-year survival are reported in **Table 2**.

**Figure 6:**
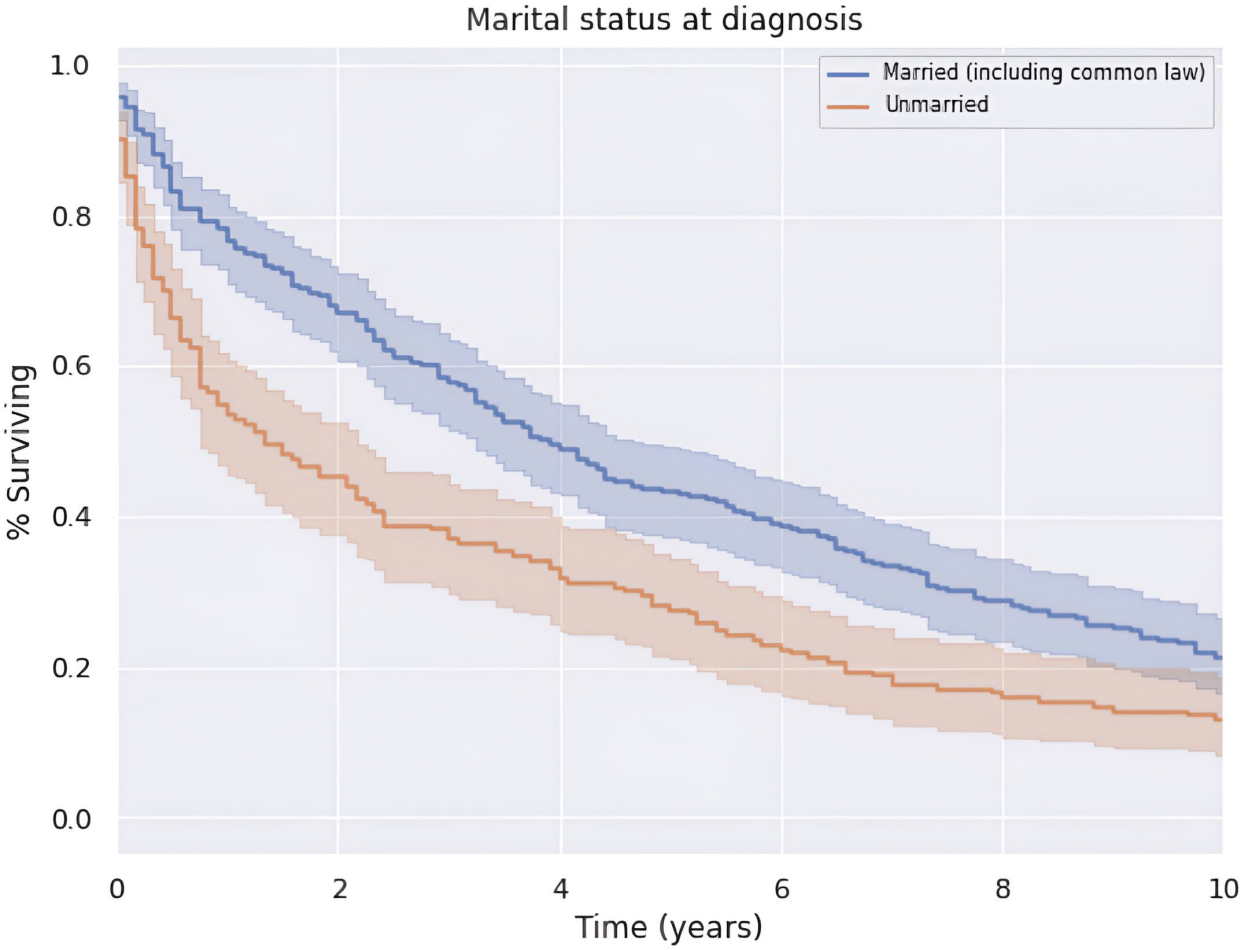
Survival by marital status at time of diagnosis.

There was an increase in survival of patients married at diagnosis compared to those who were unmarried at diagnosis (*p* < 0.05), as shown in **Figure 6**. The difference was no longer significant at approximately 9 years after diagnosis. Patients married at diagnosis had a median OS and 5-year survival of 47.0 (CI: 39.0–55.0) and 43.3%, compared to the decreased values of 16.0 (CI: 8.0–24.0) and 28.1%, respectively, for patients not married at diagnosis. This difference was not explained by differences in patient age or clinical stage at the time of diagnosis between married and unmarried men.

### 3.4 Discussion

Non-SCC of the penis is rare. Previous literature mostly consists of case reports and single-institution studies that describe particular instances of these malignancies (23–27). A large national database such as SEER, therefore, provides comprehensive data to study these tumors. The SEER database contains cancer data across various institutions in the U.S. and surveys a large percentage of the U.S. population. Past studies that utilized the SEER database to investigate non-SCC lack an updated query of the database such that contemporary (e.g., reflective of current trends of undetectable HIV viral loads and HPV vaccination) and generalizable (e.g., of sufficient sample size to allow more detailed subgroup analysis) results can be drawn (14, 15). Our study provides an updated report on the characteristics and treatment of these pathologies using the most recent cohort of non-SCC patients.

#### 3.4.1 Treatment

Our analysis emphasizes the important role of surgical intervention in treatment. Patients who underwent surgery had a better OS across the entire period observed; however, this survival advantage appears to be greatest in the first 5 years after diagnosis. Although surgery confers a survival benefit, a significant proportion of patients did not undergo surgery. The underutilization of surgical intervention may be explained by the unresectability of the tumor or late disease presentation. However, if this underutilization is due to a lack of guideline-based approach in evaluating these tumors, our findings suggest that it may be advantageous to expand surgical candidacy when feasible. The potential late disease presentation of this uncommon malignancy also emphasizes the necessity of more adequate screening guidelines for this understudied patient population, as surgery is an optimal treatment modality.

Systemic therapy or radiation, used independently or in conjunction with surgery, was utilized in a small proportion of patients. Since there is a non-significant difference in survival during the observed period between patients who did and did not receive radiation or systemic therapy, it cannot be ascertained whether these two treatment pathways are beneficial. Alternatively, it may be the case that radiation or systemic therapy is used in refractory cases where surgery is not advised or is unsuccessful.

#### 3.4.2 Histology

This analysis aims to highlight the survival characteristics with respect to a particular histological grouping. This information may be prognostically important for patients with one of these particular tumor subtypes.

Of the most prevalent histological groupings (**Table 2**), blood vessel tumors, which overwhelmingly consist of KS, had the greatest median OS and 5-year survival. This is in contrast to the previous study by Bhambhvani et al., which found KS to have a significantly worse prognostic outcome when compared to other non-SCC cancers of the penis; it should be noted, however, that this previous work stated the possibility that their results may not represent the present therapeutic landscape (14). As they acknowledged, the severity of KS corresponds with the patient’s level of immunosuppression; therefore, patients with HIV-induced immunosuppression tend to have more severe disease (28, 29). Given that highly active antiretroviral therapy (HAART) has dramatically reduced the severity of immunosuppression caused by HIV, fewer patients diagnosed with HIV are developing KS, an AIDS-defining illness. As a result of these treatments, the KS patient pool consists of those with better immune status, offering this population improved prognostic outcomes compared to other cancers (30, 31). Given that their study includes patients before 2000, when HAART was not widely available, their results do not reflect the current prognostic trend for patients with KS; our present study, thus, not only validates the previous study’s concern regarding HAART changing the prevalence and prognosis of tumors such as KS of the penis but, furthermore, describes and quantifies the current landscape of disease survival, prognosis, and Stage at the time of diagnosis in a time period with greater access to modern antiretroviral treatments that more accurately reflects the present-day clinical context.

Investigating the other common tumor types, we found basal cell neoplasms and melanomas to be two of the most populated groups with comparable median OS and 5-year survival rates of just over 40%, a significant decrease from the average 5-year survival of approximately 80% for SSC of the penis (32). The presence of these UV-dependent tumors on classically unexposed organs such as the penis highlights the importance of thorough dermatologic evaluations by physicians, including examinations of the genitalia.

Unspecified neoplasms and epithelial neoplasms, NOS had the lowest median OS and 5-year survival amongst the most prevalent subtypes. While both of these groups are classified as single categories in the SEER database, it is essential to note that they may represent a wide range of tumor types and may have different clinical characteristics and prognoses. However, epithelial neoplasms, NOS consists of carcinomas, not of squamous cell origin, and unspecified neoplasms consist of any malignant tumor that was not specified and, therefore, put in this category. These classifications may be the result of a lack of identifying microscopic features that could be obtained from the underlying histological sample; a brief analysis investigating grade suggested difficulties in identification were not related to these tumors being relatively undifferentiated (33, 34). This hinders our ability to interpret the data meaningfully and may be cause for physicians to specifically classify neoplasms, when possible, especially when rare.

#### 3.4.3 Stage and grade

Most of the patients in our cohort had localized tumors (for lymphomas, localized was defined as Stage I, IE, or IS; for non-lymphomatous cancers, localized was defined as existing solely in the tunica albuginea, an invasive tumor limited to subepithelial connective tissue, but not involving corpus spongiosum or cavernosum, or, if a skin cancer, limited to skin of the penis, prepuce, and/or the glans), which afforded them an increased survival benefit compared to those with tumors at worse stages. The rest of the patients had regional, distant, or unknown/unstaged tumors. Regional and unstaged tumors had similar prognostic indicators and tumors with distant metastases had the worst prognosis, with none of the patients living beyond 5 years. These survival characteristics are generally intuitive and consistent with literature about prognosis with respect to tumor Stage (35–37).

The tumor grade is unknown for 68% of patients; however, there were patients who fell into each category of tumor grade. Of the tumors characterized with grades I–IV, grade I tumors had the best prognosis, followed by grade II tumors. Grades III and IV afforded the worst prognosis. This is consistent with literature that suggests that decreased tumor differentiation is associated with worse outcomes (36, 38).

#### 3.4.4 Patient demographics

The results indicate that age was a determining factor in regard to the chance of survival during the observation period. Patients below 70 years had superior survival rates during the entire observation period.

Since Black patients had lower median OS and 5-year survival in comparison to White patients, it was hypothesized that Black patients were diagnosed at a later age. However, 46.7% of Black patients were diagnosed at 70 years or older compared to 51.6% for White patients. Multivariate analysis revealed statistically significant independent contributions of race, stage, marital status, and stage, as well as surgery and positive regional LN on OS (**Supplementary Table S2**).

This suggests that there are other factors responsible for the worse prognosis for Black patients; although there were trends among survival differences between White and Black patients, such as black patients demonstrating worse survival after receiving beam radiation, and worse survival upon increased disease Stage, these variables were unable to fully explain the survival gap. Further studies are needed to analyze differences in treatment with regard to race for patients with non-SCC of the penis.

The cohort is nearly split in half with respect to marriage status at diagnosis, which is reflective of the U.S. population where 53% of adults are married (39). Given the survival advantage over the 10-year period for married patients compared to unmarried patients, marriage appears to be a protective factor with respect to the survival of a penile non-SCC diagnosis, although this has been observed with penile cancer overall, to our knowledge this represents the first observation of this trend in non-SCC of the penis histologies as well (40). This trend may be attributable to the positive aspects of the marriage dyad such as better health behaviors and social support that are associated with a lower allostatic load (41). In this way, it is consistent with sociological literature that suggests that marriage affords an overall health advantage to men in the form of increased life expectancy and active life expectancy (42, 43). This is particularly relevant given that our entire cohort consists of men.

There is little evidence to support the notion that marriage can decrease the likelihood of being diagnosed with cancer. There is, however, evidence to suggest that social support in the form of marriage can have a significant impact on cancer detection and obtaining adequate treatment (44). This evidence presents a limitation of the argument that marriage itself confers a survival benefit through a decrease in the patient allostatic load, suggesting instead it may confer a benefit through early detection and treatment acquisition and adherence. Due to the rarity of penile non-SCC, it is unknown whether this limitation applies to the results of this cohort.

#### 3.4.5 Limitations

This study has limitations to note. Due to the rarity of this disease, the relatively small sample size frequently limited the power of statistical analysis. Another limitation involves the potential inclusion of cancers of squamous cell origin, which were intended to be excluded from this study. As some histologies were unable to be pathologically identified, squamous cell tumors may have been inadvertently included in the analysis. The possibility that such tumors may be of very high grade and, thus, dedifferentiation, which may prevent pathological confirmation, was considered; however, no trend between tumor grade and the population of unidentifiable histologies was qualitatively observed.

This study also faced challenges regarding treatment paradigms. Due to the lack of specific data within the SEER database, it cannot be distinguished whether radiotherapy was performed at the primary site or nodal site(s) nor are precise chemotherapeutic regimens recorded. The inability to analyze these treatment nuances limits what we can understand about treatment effectiveness.

The heterogeneity of histological types of non-SCC penile cancer also makes it challenging to develop and analyze specific treatment paradigms. For example, while surgery was a beneficial treatment modality as a whole, this is likely due to the cohort mostly consisting of epithelial neoplasms; other tumor types may not benefit from surgery. As such, while our results may give a broad overview, they may not apply to all histological subtypes.

Age is a potential confounding factor that could have influenced the results. While age was considered in our multivariate analysis, it can influence the rest of the variables not selected for the multivariable analysis. Age can affect the patients’ overall health, their ability to tolerate treatment, the presence of comorbidities, and their access to and utilization of healthcare resources.

Finally, it should be noted that the SEER database encompasses data from American patients only. While the SEER database provides a comprehensive collection of cancer statistics, the demographic makeup of the U.S. differs considerably from other regions in the world. This is especially relevant considering the global importance and varied geographical distribution of penile cancer.

## 4 Conclusions

This study queried a large-scale population database to investigate the demographics, clinical characteristics, and outcomes of patients with non-SCC of the penis. These tumors are rare and extremely diverse. Consequently, survival rates are variable and dependent on numerous factors such as histology and tumor Stage. Overall, the median OS is relatively moderate at 3.2 years, and the 5-year survival is low at 37.7%. Similarly, these results underscore the importance of timely urologic evaluation and surgical intervention. Although systemic therapy and radiation were uncommonly pursued treatment pathways in this cohort, providers and patients may engage in shared decision making when considering all available treatment options. Our study highlights common characteristics, treatment offerings, and outcomes of penile non-SCC. Future studies should continue to investigate penile non-SCC to ensure that patients are connected with the right treatment at the right time and reduce health inequities in penile cancer care.

## Data availability statement

The original contributions presented in the study are included in the article/**Supplementary Material**. Further inquiries can be directed to the corresponding author.

## Ethics statement

Ethical approval was not required for the study involving humans in accordance with the local legislation and institutional requirements. Written informed consent to participate in this study was not required from the participants or the participants’ legal guardians/next of kin in accordance with the national legislation and the institutional requirements.

## Author contributions

LA: Formal Analysis, Investigation, Writing – original draft. KS: Writing – original draft, Writing – review & editing. AJ: Funding acquisition, Writing – review & editing. GE: Formal Analysis, Investigation, Writing – original draft, Writing – review & editing. MP: Writing – review & editing. AB: Conceptualization, Data curation, Formal Analysis, Investigation, Methodology, Project administration, Software, Supervision, Validation, Visualization, Writing – review & editing.
