# Supplementary material for: A SEER database retrospective cohort of 547 patients with penile non-squamous cell carcinoma: demographics, clinical characteristics, and outcomes
Source: Front Oncol. 2023 Oct 31;13:1271913. doi: 10.3389/fonc.2023.1271913 (PMC10644775; doi:10.3389/fonc.2023.1271913)
Supplement: Supplementary file 1 [file Table_1.docx]

Supplemental Table 1

|  | Adjuvant Therapy & Stage | Adjuvant Therapy & Grade |
| --- | --- | --- |
| **Concordance** | 0.78 | 0.70 |
| **Partial AIC** | 387.19 | 411.92 |
| **log-likelihood ratio test** | 35.50 on 2 df | 10.76 on 2 df |
| **-log2(p) of ll-ratio test** | 25.61 | 7.76 |

Supplemental Table 2: Multivariate Analysis for Demographic and Treatment Variables

|  | coef | z | p | -log2(p) |
| --- | --- | --- | --- | --- |
| Reason no cancer-directed surgery | 2.22 | 3.21 | <0.005 | 9.54 |
| Chemotherapy recode (yes, no/unk) | -0.78 | -0.86 | 0.39 | 1.35 |
| Radiation recode | 0.68 | 0.84 | 0.4 | 1.32 |
| Regional nodes examined (1988+) | -5.23 | -2.56 | 0.01 | 6.57 |
| Regional nodes positive (1988+) | 5.75 | 3.18 | <0.005 | 9.43 |
|  |  |  |  |  |
| Race recode (W, B, AI, API) | 3.08 | 4.23 | <0.005 | 15.38 |
| Combined Summary Stage (2004+) | 2.68 | 4.07 | <0.005 | 14.36 |
| Age recode with <1 year olds | -7.41 | -3.32 | <0.005 | 10.12 |
| Marital status at diagnosis | 4.39 | 5.16 | <0.005 | 21.9 |
